# Supplementary material for: A Global Screen for Assembly State Changes of the Mitotic Proteome by SEC-SWATH-MS
Source: Cell Syst. 2020 Feb 26;10(2):133–155.e6. doi: 10.1016/j.cels.2020.01.001 (PMC7042714; doi:10.1016/j.cels.2020.01.001)

Q969X5 | ERGI1_HUMAN | ERGIC1 ERGIC32 KIAA1181 HT034
Monomer MW [kDa]: 32.592 Monomer expected elution fraction: 50

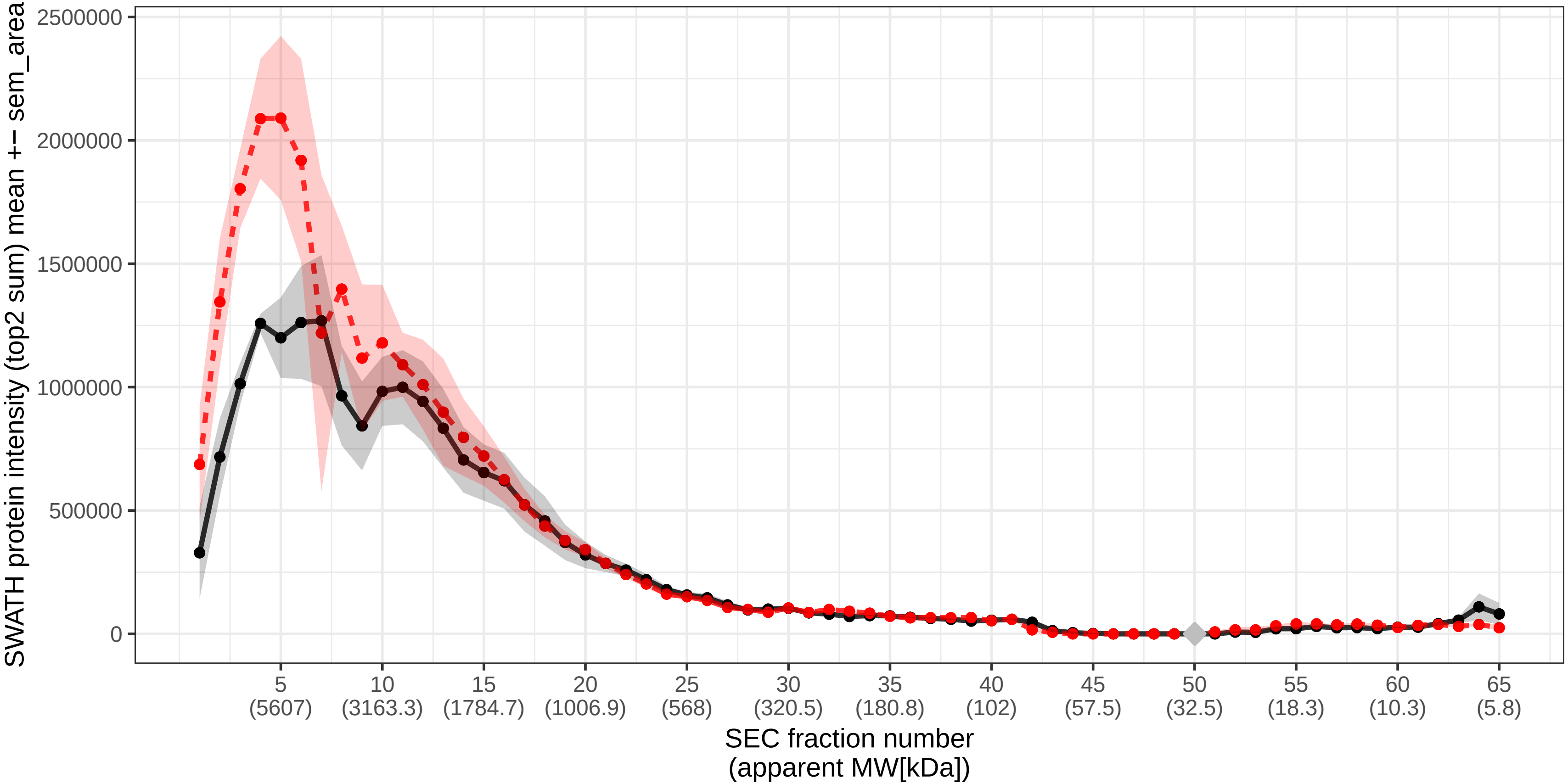

Supplement: Data S1. SEC-SWATH-MS Protein Chromatograms, Related to Figure 1 [file mmc6.zip › SECchrom_Q969X5_ERGI1_HUMAN_ERGIC1_ERGIC32_KIAA1181_HT034.pdf]
